# Age, Period, and Cohort Effects on Suicide Mortality in South Korea, 1992–2015

**DOI:** 10.3390/ijerph15081580

**Published:** 2018-07-25

**Authors:** Soonjoo Park, Yeong-Jun Song, Jinseob Kim, Myung Ki, Ji-Yeon Shin, Young-Man Kwon, Jiseun Lim

**Affiliations:** 1 College of Nursing, Eulji University, Daejeon 34824, Korea; sjpark@eulji.ac.kr; 2 Department of Preventive Medicine, Eulji University School of Medicine, Daejeon 34824, Korea; syjace@nate.com; 3 Laboratory of Genome Epidemiology and Health Big Data, Graduate School of Public Health, Seoul National University, Seoul 08826, Korea; secondmath85@gmail.com; 4 Department of Preventive Medicine, College of Medicine, Korea University, Seoul 136-701, Korea; myungki@korea.ac.kr; 5 Department of Preventive Medicine, School of Medicine, Kyungpook National University, Daegu 41944, Korea; jyshin@knu.ac.kr; 6 Department of Medical IT and Marketing, College of Health Industry, Eulji University, Seongnam 34824, Korea; ymkwon@eulji.ac.kr

**Keywords:** suicide, age, period, and cohort effects, identification problem, Korea

## Abstract

Although the effects of age, period, and cohort (APC) on suicide are important, previous work in this area may have been invalid because of an identification problem. We analyzed these effects under three different scenarios to identify vulnerable groups and thus overcame the identification problem. We extracted the annual numbers of suicides from the National Death Register of Korea (1992–2015) and estimated the APC effects. The annual average suicide rates in 1992–2015 were 31.5 and 14.7 per 100,000 males and females, respectively. The APC effects on suicide were similar in both sexes. The age effect was clearly higher in older subjects, in contrast to the minimal changes apparent during earlier adulthood. The birth cohort effect showed an inverted U shape; a higher cohort effect was evident in females born in the early 1980s when period drift was larger than 3.7%/year. Period effect increased sharply during the early 1990s and 2000s. We found that elderly and young females may be at a particularly high risk of suicide in Korea.

## 1. Introduction

Recently, the suicide rate in South Korea (hereinafter, Korea) has increased greatly. At about fourfold over the past two decades, this rate is the highest among all Organization for Economic Co-operation and Development (OECD) countries since the early 2000s [1]. Although the 1997–1998 Asian financial crisis triggered an increase in suicide, especially among Korean males aged 35–64 years, a dramatic and persistent increase in the suicide rate of the elderly seems to have played a major role in the recent epidemic. The suicide rates in 2014 were 105 and 230 per 100,000 Koreans aged 70–74 and >85 years, respectively [2,3]. The relevant sociocultural factors and underlying causes of the greater vulnerability to suicide among the elderly remain poorly understood. The patterns and characteristics of suicide should be determined before establishing suicide-prevention strategies. Especially in terms of the elderly, distinguishing between age and cohort effects is important to understand the causes of suicide and ensure that appropriate support is available. Some recent studies found that both increasing age effect and strong cohort effects increased the risk of suicide of the elderly in Korea [4,5]. Previous Korean studies not using age, period, and cohort (APC) analysis have suggested that higher suicide rate in the elderly may be attributable to poor physical condition and insufficient social security, and recent increase of suicide rate may be associated with economic recession and weakened social integration [6,7,8,9,10,11].

Many researchers worldwide (including Koreans) have sought to identify the APC effects of suicide. However, an inherent identification problem compromises the validity of the results. Several studies found that no known statistical process reliably estimates true APC effects, including the intrinsic estimator (IE) widely applied in recent investigations [12,13]. Therefore, some studies attempted to overcome the identification problem by obtaining nonlinear APC shapes after setting the slopes of the cohort and period effects to zero, or by exploring APC effects according to specific constraints [14,15,16]. In the present study, we evaluated the effects of APC on the suicide rate after making several assumptions based on prior knowledge. We used the shapes of nonlinear APC components to identify vulnerable groups and predict future suicide trends. 

## 2. Materials and Methods

### 2.1. Data

We extracted data on the annual number of suicides and resident registration population to 1 July for all years between 1991 and 2015, from the National Death Registration and Vital Statistics Survey of Statistics Korea, respectively. Suicide is coded as X60–X84 in the 10th revision of the International Classification of Diseases (ICD-10) [17]. We retrieved data on age at suicide (age), birth year (cohort), and the year of suicide (period). The age at suicide and the year of suicide were collected based on the certification of death, and the birth year was calculated by the following equation: birth year = year of suicide − age at suicide. This study was approved by the Institutional Review Board of Eulji University, Korea (EU15-08).

### 2.2. Analysis

We calculated suicide age-specific and age-standardized rates (ASRs). To calculate age-specific rates, we categorized age as 10–14, 15–19, 20–24, 25–49, 50–64, 65–79, and ≥80 years. ASRs were calculated according to five-year age groups; the national population in 2005 served as the standard. To overcome the identification problem, we applied the concept of drift (the sum of the linear effects of period and cohort) [18]. This value does not change when different proportions of the drift are attributed to cohort or period [18]. The exact portions of drift included in period and cohort effect cannot be determined statistically. Therefore, we arbitrarily set a linear period effect (hereafter, period drift, PD) and estimated the APC effects using the method of Carstensen [19].

The PDs were chosen based on the following two assumptions: (1) Suicide risk in those aged ≥60 years was greater than that in those aged 40–60 years, after adjustment for cohort and period effects. This is due to the fact that being elderly per se is a risk factor for suicide in Korea; a higher suicide rate in the elderly is associated with afflictions of old age (comorbid chronic diseases, activity limitations, and insufficient social security), and thus not with old generation [9,10]; and, (2) the general period effect over the past two decades after adjusting for age and cohort effects would be positive. Many studies have concluded that the recent status of poor economy, deepened socioeconomic inequality and weakened social integration is associated with the recent increase in suicide rates [6,7,8,11]. A PD of 0%/year assumes that the linear period effect is zero. A PD of 7.4%/year was visually confirmed, validating the assumption that suicide risk in those aged ≥60 years was approximately equal to that in adults aged 40–60 years (for both genders). We present the APC effects associated with PDs of 3.7%/year positioning in the middle of two graphs relevant to the assumptions described above. 

We fitted natural spline graphs with ten knots for each component of APC to detect non-linear effects. We set the 1958 birth cohort as the reference cohort. The age effects are interpretable as age-specific rates of 1958 birth cohort after adjustment for the period effect [19]. The cohort and period effects are interpretable as rate ratios relative to reference cohort and rate ratios relative to the age-cohort prediction, respectively [19]. APC analysis was performed using the *apc.fit* function of the Epi package of R software (ver. R.3.0.2; R Foundation for Statistical Computing, http://www.r-project.org). All analyses were performed by gender, given that suicide rates differed by APC between males and females. 

## 3. Results

### 3.1. Trends in Suicide Rates

**Figure 1:**
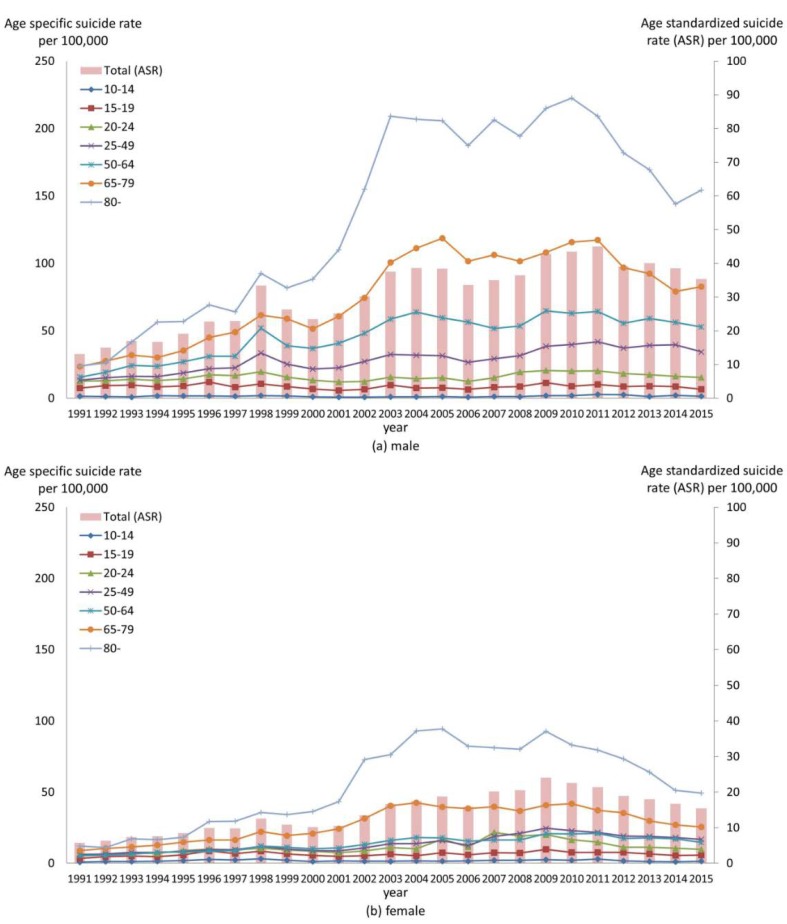
Age-specific and -standardized suicide rates per 100,000 males (**a**) and females (**b**) in 1991–2015.

In 1992–2015, the total suicide numbers and average annual suicide rates were 165,626 and 31.5 per 100,000 for males and 77,454 and 14.7 per 100,000 for females. The age-standardized suicide rate increased since 1991, with waxing and waning patterns in males and females. It was at its highest in 2011 for males (45.0 per 100,000) and in 2009 for females (24.0 per 100,000), and it showed a prominent peak in 1998 for males. For the elderly (≥65 years of age), incomparable increases in suicide rates were observed over the period 2000–2005 and continued to increase up to 2010 in both males and females. A dramatic increase in 1998, after the economic crisis, was evident especially in males aged 25–64 years and in males and females older than 65, and the high suicide rate persisted thereafter (Figure 1). 

### 3.2. APC Effects

**Figure 2:**
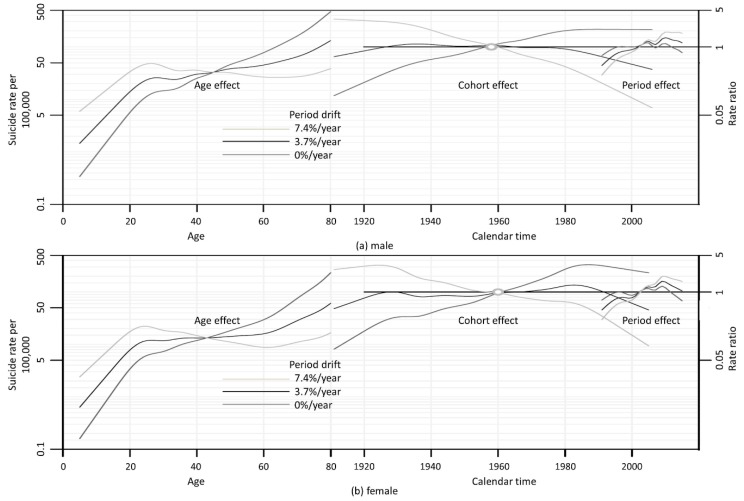
Age, period, and cohort (APC) effects on suicide for males (**a**) and females (**b**). The y-axes indicate suicide rates per 100,000 persons by age, and rate ratios by the birth cohort and period.

The overall APC drifts (the sums of the cohort and PDs) were 3.7% and 4.8%/year in males and females, respectively (Figure 2). We drew three graphs with different PD values (0%, 3.7% and 7.4%/year) to explore the APC effects. The age effects are plotted on the left y-axis (suicide rates per 100,000 of the population); the period and cohort effects are plotted on a rate ratio scale (right axis). 

The age effects during childhood and adolescence consistently increased, independent of the PD, in both genders. Various linear trends of age effects were evident during adulthood and old age, i.e., an increase (at PD = 0% and 3.7%/year) or a slight decline (at PD = 7.4%/year) during adulthood, and a moderate (at PD = 7.4%/year) or steep (at PD = 0% and 3.7%/year) increase during old age (>72 years of age in males and >64 years of age in females). The increase in the age effect during old age did not change even when the PD was >7.4% (data not shown).

Independent of the PD, an inverted U-shaped relationship was evident between birth cohort and suicide in both males and females. Various linear trends in the birth cohort effect were evident at the three PDs; overall, suicide risk increased, unchanged, or decreased in recent generations at PDs of 7.4%, 3.7% and 0%/year, respectively. In males, the suicide risk increased or remained unchanged in those born before 1938; increased, decreased, or remained unchanged in those born in 1938–1987; and decreased or was unchanged in those born later. In females, the suicide risk in those born before 1928 increased or remained unchanged; increased, decreased, or remained unchanged in those born in 1928–1983; and decreased in those born thereafter; two small peaks were apparent around 1928 and 1983 (See Supplementary Materials for Table S1 of birth cohort effect). Period effects increased sharply since 1991, but the increase slowed in the mid-1990s. After the early 2000s, period effects again increased steeply, but decreased after 2010 in males and after 2009 in females (Figure 2).

## 4. Discussion

In this study, we identified the APC effects on suicide risk, which were similar for both genders: (1) The age effect clearly increased after entering old age, in contrast to the relatively minimal change seen during adulthood; (2) the birth cohort effect had an inverted U shape; and, (3) a sharp increase in the period effect was evident during the early 1990s and 2000s. 

In general, the age effects of most health problems are similar across populations, but in the case of suicide, age effects vary by the sociocultural environment. For example, age effects after adulthood vary among nations. A higher risk of suicide in old age than in adulthood may reflect social isolation, poor physical or financial condition, or an increased risk of successful suicide in those who have repeatedly shown suicidal behavior [20,21,22]. On the other hand, a higher risk of suicide in adulthood than old age may be attributable to perceived burdensomeness related to achieving tasks during social life or physical change such as menopause [5,23,24]. The age effect is determined by the more influential of the two situations described in the context of a particular society and culture. 

Consistent with our findings of clear increases in the age effect during childhood and adolescence, previous studies also reported steep increases during these periods [4,5,14,15,23,25]. However, the age effects during adulthood and old age vary by nation. For example, the age effect peaks at around 50 years in Japan [5], while in Switzerland it increases sharply during middle age and continues into old age [14]. Here, we found a consistent increase in the age effect during old age independent of the PD, in line with the fact that Korea has both the highest elderly poverty rate and the widest income gap between working families and the elderly [26]. The risk of suicide in the elderly increases when multiple chronic illnesses and financial stress, poor social policies and an inadequate welfare system combine to create a crisis. Moreover, social isolation, recently aggravated by increased divorce rates among older adults, may trigger suicide [27]. A suicide prevention program targeting the physical, financial, and social challenges encountered by the elderly is essential.

In this study, the birth cohort effect on suicide showed an inverted U shape for both males and females. The increase in suicides after 2000 in males aged >65 years and females aged >80 years (Figure 1) may be partly attributable to the increasing cohort effect to 1938 in males, and to 1928 in females in combination with the increased age effects during old age. Also, a peak in the cohort effect in females in the early 1980s was evident at PDs ≥3.7%/year, suggesting that females born in the early 1980s may be vulnerable to suicide. Empirical studies suggested that suicidality was closely associated with parental rearing behaviors [28,29,30]; the observed vulnerability to suicide may be associated with the characteristics of parents born during the Korean War (1950–1953). Previous studies suggested that unfavorable social and economic conditions affect males more strongly than females, whereas parental rearing was more associated with suicidality in females than males [31,32]; this is in line with our observation of heightened vulnerability to suicide only in the young female generation. If Korean females born in the early 1980s are indeed especially vulnerable to suicide, the suicide rate would be expected to be extremely high during old age in these individuals, although higher suicide rate is not apparent currently, because these females are presently aged 30–40 years. Thus, these females should be monitored, in terms of mental health, suicidal behaviors, and suicide per se, for many years into the future. 

We found that the period effect started to increase in 1991, that is., before the economic crisis. Previous studies explored the recent rapid increase in suicide rates from an economic perspective, or in the context of poor social integration [7,8,11,33]. A previous study showed that the increasing suicide rate in Korea during 1983–2002 was associated with marriage and divorce rates [8]. Another previous study using time-trend analysis suggested that for males, the unemployment rate was more important in terms of suicide from 1985–2006 than the divorce or marriage rate [11]. The increasing period effect evident since 1991 may have given rise to both unstable economic conditions and social disintegration; these two factors may affect different groups differently. 

The period effect either did not change or declined in the late 1990s and then increased again in the early 2000s. This recent rise reflects decreasing social integration up to the mid-2000s and celebrity suicides occurring in the period 2005–2009 [34,35]. Afterward, the trend declined among women first in 2010, and was followed by men in 2012. An explanation for the time gap of decreasing suicide rates between men and women would be the lower rate of unemployment after 2010 and the 2011 paraquat prohibition [36,37], which influenced men more significantly [11,38]. However, no secular trend, or combination of trends, fully explains the trend in the period effect; thus, further studies are necessary. To summarize the age and period effects seen in this study, the suicide rate would likely decline for some time if the recent falls in the period and birth cohort effects persist. However, as Korea is changing greatly in cultural, economic, and political terms, short-term variations in the APC effects are possible; continuous and careful nationwide monitoring of these effects is essential.

### Limitations and Strengths

Our study had certain limitations. First, the Korean suicide data may be incomplete, because many undetermined deaths (deaths with codes of Y10–Y34 in ICD-10) and accidental deaths (e.g., accidental poisoning: deaths with codes of X40–X49 in ICD-10) could have been suicides. Because the quality of the death statistics has improved since 1999, and a substantial number of accidental deaths are presumed to have been categorized to suicide recently in Korea, bias in APC analyses cannot be ruled out in this study [39]. The APC identification problem constituted the second limitation. Here, we presented the range of APC effects rather than point estimations; this renders the interpretation of linear APC trends more difficult. Many previous studies derived point estimations of the APC effects using various statistical methods, including IE, to clearly define recent trends. However, no statistical method, including IE, accurately deals with all three effects; therefore, there is an identification problem. Thus, we used ranges rather than point estimations to derive valid results. In a sense, this limitation is in fact the unique strength of our study. A few previous works dealt with the APC effects by arbitrarily setting PD values [16]. We used an alternative method to focus on nonlinear components to deal with the APC identification problem. Third, this study investigated a relatively short time period from 1992 to 2015. Therefore, young, middle, and old generations were used to estimate only the age effect during young, middle, and old age, respectively, which may have weakened the validity of our estimates for the APC effects. 

## 5. Conclusions

In conclusion, elderly and young female generation may be at a high risk of suicide. Careful nationwide surveillance and continuous monitoring of the APC effects are necessary.

## Supplementary Materials

The following are available online at http://www.mdpi.com/1660-4601/15/8/1580/s1, Table S1. The birth cohort effects on suicide for males and females according to three period drift.
