# Supplementary material for: Age, Period, and Cohort Effects on Suicide Mortality in South Korea, 1992–2015
Source: Int J Environ Res Public Health. 2018 Jul 25;15(8):1580. doi: 10.3390/ijerph15081580 (PMC6121370; doi:10.3390/ijerph15081580)
Supplement: Supplementary file 1 [file ijerph-15-01580-s001.zip › ijerph-321505-SI.pdf]

Table S1. The birth cohort effects on suicide for males and females according to three period drift

Birth year	Male			Female		
	Period drift (%/year)					
	0.0	3.7	7.4	0.0	3.7	7.4
1911	0.12	0.65	3.38	0.08	0.48	2.68
1912	0.13	0.67	3.36	0.09	0.51	2.72
1913	0.13	0.69	3.33	0.10	0.53	2.77
1914	0.14	0.71	3.31	0.11	0.56	2.82
1915	0.15	0.73	3.29	0.12	0.59	2.86
1916	0.16	0.75	3.26	0.13	0.62	2.91
1917	0.17	0.77	3.24	0.14	0.66	2.96
1918	0.19	0.79	3.22	0.15	0.69	3.01
1919	0.20	0.81	3.20	0.16	0.73	3.06
1920	0.21	0.84	3.17	0.18	0.77	3.11
1921	0.22	0.86	3.15	0.20	0.80	3.16
1922	0.24	0.89	3.13	0.21	0.84	3.20
1923	0.26	0.91	3.11	0.23	0.88	3.23
1924	0.27	0.94	3.08	0.25	0.92	3.25
1925	0.29	0.96	3.06	0.27	0.95	3.24
1926	0.31	0.99	3.03	0.28	0.98	3.21
1927	0.33	1.01	3.00	0.30	0.99	3.16
1928	0.35	1.03	2.96	0.31	1.00	3.08
1929	0.37	1.06	2.92	0.33	1.00	2.97
1930	0.39	1.08	2.87	0.33	0.99	2.83
1931	0.41	1.09	2.82	0.34	0.97	2.67
1932	0.43	1.11	2.76	0.34	0.93	2.49
1933	0.45	1.12	2.69	0.34	0.90	2.32
1934	0.47	1.13	2.62	0.34	0.87	2.16
1935	0.49	1.13	2.54	0.34	0.84	2.01
1936	0.51	1.13	2.45	0.34	0.82	1.90
1937	0.53	1.13	2.36	0.35	0.81	1.81
1938	0.55	1.13	2.27	0.36	0.81	1.75
1939	0.56	1.12	2.18	0.38	0.81	1.70
1940	0.58	1.11	2.09	0.40	0.82	1.66
1941	0.59	1.10	1.99	0.42	0.84	1.63
1942	0.61	1.09	1.90	0.44	0.85	1.59
1943	0.62	1.07	1.82	0.46	0.85	1.55
1944	0.64	1.06	1.73	0.48	0.86	1.50
1945	0.66	1.05	1.66	0.50	0.86	1.45
1946	0.67	1.04	1.59	0.51	0.85	1.39
1947	0.69	1.03	1.52	0.53	0.85	1.33
1948	0.72	1.03	1.46	0.54	0.84	1.28

Table S1. The birth cohort effects on suicide for males and females according to three period drift (continued)

Birth year	Male			Female		
	Period drift (%/year)					
	0.0	3.7	7.4	0.0	3.7	7.4
1949	0.74	1.03	1.41	0.56	0.83	1.22
1950	0.77	1.03	1.36	0.58	0.83	1.18
1951	0.80	1.03	1.32	0.59	0.83	1.13
1952	0.83	1.04	1.28	0.62	0.83	1.09
1953	0.87	1.04	1.24	0.65	0.83	1.06
1954	0.91	1.05	1.21	0.68	0.84	1.04
1955	0.95	1.05	1.17	0.72	0.86	1.03
1956	0.98	1.06	1.14	0.76	0.88	1.01
1957	1.02	1.06	1.10	0.81	0.90	1.00
1958	1.06	1.06	1.06	0.86	0.92	0.99
1959	1.10	1.06	1.03	0.91	0.94	0.98
1960	1.14	1.06	0.99	0.96	0.96	0.96
1961	1.17	1.05	0.95	1.01	0.97	0.94
1962	1.21	1.04	0.91	1.05	0.98	0.91
1963	1.23	1.03	0.86	1.09	0.98	0.88
1964	1.26	1.01	0.82	1.14	0.99	0.86
1965	1.28	0.99	0.78	1.19	0.99	0.83
1966	1.31	0.98	0.74	1.24	1.00	0.81
1967	1.35	0.97	0.71	1.30	1.01	0.79
1968	1.39	0.97	0.68	1.36	1.02	0.77
1969	1.44	0.97	0.66	1.43	1.03	0.75
1970	1.50	0.97	0.64	1.51	1.05	0.74
1971	1.55	0.97	0.61	1.58	1.06	0.72
1972	1.61	0.97	0.59	1.67	1.08	0.71
1973	1.67	0.97	0.57	1.76	1.10	0.69
1974	1.73	0.97	0.55	1.86	1.12	0.68
1975	1.79	0.97	0.53	1.97	1.14	0.67
1976	1.85	0.96	0.51	2.09	1.17	0.67
1977	1.90	0.95	0.49	2.23	1.21	0.66
1978	1.95	0.94	0.47	2.39	1.24	0.66
1979	1.99	0.93	0.44	2.55	1.28	0.66
1980	2.03	0.91	0.42	2.72	1.31	0.65
1981	2.06	0.89	0.40	2.87	1.34	0.64
1982	2.09	0.87	0.38	3.02	1.36	0.63
1983	2.11	0.85	0.35	3.13	1.36	0.61
1984	2.13	0.83	0.33	3.22	1.35	0.58
1985	2.14	0.80	0.31	3.28	1.32	0.55
1986	2.15	0.78	0.29	3.31	1.29	0.52

Table S1. The birth cohort effects on suicide for males and females according to three period drift (continued)

Birth year	Male			Female		
	Period drift (%/year)					
	0.0	3.7	7.4	0.0	3.7	7.4
1987	2.16	0.75	0.27	3.32	1.25	0.48
1988	2.16	0.73	0.25	3.31	1.20	0.45
1989	2.16	0.70	0.24	3.28	1.14	0.41
1990	2.16	0.68	0.22	3.24	1.09	0.38
1991	2.16	0.65	0.20	3.18	1.03	0.35
1992	2.16	0.63	0.19	3.12	0.98	0.32
1993	2.16	0.60	0.18	3.05	0.92	0.29
1994	2.15	0.58	0.16	2.99	0.87	0.26
1995	2.15	0.56	0.15	2.92	0.82	0.24
1996	2.15	0.54	0.14	2.85	0.77	0.22
1997	2.15	0.52	0.13	2.79	0.73	0.20
1998	2.15	0.50	0.12	2.73	0.69	0.18
1999	2.15	0.48	0.11	2.67	0.65	0.16
2000	2.14	0.47	0.11	2.61	0.61	0.15
2001	2.14	0.45	0.10	2.55	0.57	0.14
2002	2.14	0.43	0.09	2.49	0.54	0.12
2003	2.14	0.42	0.09	2.44	0.51	0.11
2004	2.14	0.40	0.08	2.38	0.48	0.10
2005	2.14	0.39	0.07	2.33	0.45	0.09
